# The effects of stenting on coronary endothelium from a molecular biological view: Time for improvement?

**DOI:** 10.1111/jcmm.13936

**Published:** 2018-10-23

**Authors:** Anne Cornelissen, Felix Jan Vogt

**Affiliations:** ^1^ Department of Cardiology, Pneumology, Angiology, and Internal Intensive Medicine University Hospital Aachen Aachen Germany

**Keywords:** (re‐) endothelialization, coronary stents, endothelial denudation, endothelial function, pro‐healing stents, restenosis, stent thrombosis

## Abstract

Coronary artery stenting following balloon angioplasty represents the gold standard in revascularization of coronary artery stenoses. However, stent deployment as well as percutaneous transluminal coronary angioplasty (PTCA) alone causes severe injury of vascular endothelium. The damaged endothelium is intrinsically repaired by locally derived endothelial cells and by circulating endothelial progenitor cells from the blood, leading to re‐population of the denuded regions within several weeks to months. However, the process of re‐endothelialization is often incomplete or dysfunctional, promoting in‐stent thrombosis and restenosis. The molecular and biomechanical mechanisms that influence the process of re‐endothelialization in stented segments are incompletely understood. Once the endothelium is restored, endothelial function might still be impaired. Several strategies have been followed to improve endothelial function after coronary stenting. In this review, the effects of stenting on coronary endothelium are outlined and current and future strategies to improve endothelial function after stent deployment are discussed.

## 1 WHAT HAPPENS TO VASCULAR ENDOTHELIUM DURING ANGIOPLASTY/STENT DEPLOYMENT?

Catheter‐based interventional strategies such as percutaneous transluminal coronary angioplasty (PTCA) and coronary stent implantation (referred to by “percutaneous coronary intervention” – PCI) represent the gold standard in revascularization of coronary artery stenoses. However, both balloon angioplasty and stent implantation inevitably lead to near complete damage and loss of endothelial cells.^1^, ^2^, ^3^ The loss of a continuous endothelial monolayer facilitates potentially lethal consequences such as thrombus formation and restenosis.

The vascular endothelium acts as a barrier between the vessel wall and the blood flow. It regulates a variety of vessel functions, including the vascular tone and the passage of macromolecules such as lipoproteins.^4^, ^5^ Cell junctions maintain the integrity of the endothelial monolayer. Tight, adherens and gap junctions establish the connection of endothelial cells to each other whereas integrins form a link to the extracellular matrix proteins (eg, fibronectin and vitronectin).^4^, ^6^

The healthy endothelium controls the tone and the proliferative state of the underlying vascular smooth muscle cells (VSMCs) by the production and release of bioactive substances as a response to changes in its physical, chemical, and humoral environment. Nitric oxide (NO) is known to be one of the most potent vasorelaxant and platelet‐inhibitory molecules and is produced by endothelial nitric oxide synthase (eNOS) from L‐arginine.^7^ The release of NO by the endothelium initiates cyclic GMP (cGMP)‐mediated relaxation of the underlying smooth muscle cells, resulting in increased blood flow and inhibition of the coagulation cascade. Nitric oxide also inhibits platelet adhesion and aggregation by inhibiting the thromboxane A2 receptor.^8^ In addition to these vasorelaxant and antithrombotic effects, endothelium‐derived NO contributes to the inhibitory effect of endothelial cells on VSMCs. An incubation of co‐cultures of VSMCs and endothelial cells with L‐arginine, the substrate of NO‐synthesis, led to a decrease of VSMC growth while this effect was decreased when cells were incubated with the L‐arginine analogue nitro‐L‐arginine, thus blocking the NO synthase.^9^ Studies performed in rabbit models of atherosclerosis^10^ and of balloon injury^11^ evidenced an inhibition of neointima formation after long‐term oral administration of L‐arginine.

In concert with NO, the significant role of the endothelium in maintaining the balance between vasoconstriction and vasodilatation is carried out by the production and secretion of vasoconstrictive substances such as endothelin, reactive oxygen species, endothelium‐derived cyclo‐oxygenase‐dependent vasoconstricting factor, prostaglandin H_2_, thromboxan A_2,_ and angiotensin II.^12^, ^13^

Thus, the functional vascular endothelium has a striking role in keeping the balance between blood coagulation and fibrinolysis and the vascular tone.^14^ Beneath its non‐thrombogenic properties that maintain the fluidity of the blood exerted by NO and other molecules such as thrombomodulin, heparin‐like molecules, and prostacyclin, prothrombotic endothelial molecules including von Willebrand factor and plasminogen activator inhibitor‐1 act as counterparts.^15^, ^16^ Endothelial cell‐derived Prostaglandin I_2_ inhibits platelet activation^17^ and therefore acts synergistically to NO that inhibits platelet adhesion and aggregation as mentioned above.^18^

Taken together, the initiation and progression of atherosclerosis is characterized by disturbances of normal endothelial functions.^19^ The endothelial dysfunction leads to an impaired vascular homeostasis, notably an impaired endothelium‐dependent vasodilation and endothelial activation, associated with a pro‐inflammatory and procoagulant environment.^20^ Particularly the bioavailability of NO is reduced, caused by enhanced degradation of NO and decreased eNOS expression.^21^ Besides the resulting impairment of vasodilation the reduced bioavailability of NO leads to apoptosis of endothelial cells, additionally triggered by local inflammatory processes.^19^ The endothelial cell turnover is accelerated, however the regenerated cells may be senescent, lack endothelial barrier integrity and may be unable to produce sufficient NO, which results in increased oxidation of LDL and further progression of atherosclerosis.^22^

Intravascular processes such as balloon angioplasty and stent implantation inevitably cause severe vascular injury as well.^23^ It has been demonstrated that balloon injury induces splitting of the atheromatous plaque and stretching of the vessel wall, notably of media and adventitia, and lysis of some of the cells, mainly VSMCs. These effects have been described both in animal models^1^, ^24^ and in human postmortem arteries^3^, ^25^ and are accompanied by important alterations in the mechanical environment of the vessel wall.^26^

On a macroscopic point of view the angioplasty immediately induces a change in the external size of the artery at the injured site, but the geometry of the vessel segment returns approximately to the 3‐D shape it had before the plaque intruded into the lumen.^27^ Initially, the effect of elastic recoil occurring seconds to hours after PTCA could be observed in 5%‐10% patients undergoing angioplasty.^28^ Additionally, local arterial injury provokes an increased local release of vasoconstrictive agents such as serotonin and thromboxane, possibly resulting in vasospasms.^29^, ^30^ Angioplasty produces endothelial denudation, resulting in a disturbance of the integrity of structures inside the diseased arterial wall^2^, ^24^ and followed by rapid platelet deposition and attraction of leukocytes.^31^, ^32^ VSMC proliferation and deposition of extracellular matrix proteins contributes to an intimal thickening, neointimal hyperplasia, and finally the development of a restenosis following PCI.^33^ The release of chemotactic factors and mitogens for VSMCs by the platelets, such as platelet‐derived growth factor (PDGF), together with the mechanical forces to the VSMCs lead to their activation, resulting in proliferation, migration and shift from a contractile to a synthetic phenotype.^34^ Only some 15 years ago, Indolfi et al demonstrated that the extent of balloon injury is directly proportional to VSMCs proliferation.^35^ After inserting a Fogarty catheter into the lumen of a rat common carotid artery, different inflation pressures of 0, 0.5, 1.0, 1.5, and 2.0 ATM were applied. Remarkably it was evidenced for the first time that the proliferative response of VSMCs was proportional to the degree of vascular injury, confirmed by histopathologic findings as well as by the extraction of RNA at 30 minutes after balloon injury, demonstrating that an increase of pressure resulted in higher *c‐fos* expression and drives neointimal formation and proliferation.

Another long‐term process has been identified as one major determinant of restenosis after balloon angioplasty in humans, known as constrictive vascular remodelling.^36^ Arterial remodelling in general represents an adaptive or compensatory response of blood vessels to hemodynamic stress, arterial injury, and cellular proliferation and can either be constrictive or dilative.^37^ Constrictive vascular remodelling may be the consequence of vessel constriction due to a retractile scar. Compensatory dilation on the other hand delays the development of focal stenosis in native atherosclerotic arteries despite significant plaque accumulation as the outer vessel diameter increases.^37^ In stented segments a compensatory dilation by increase of the outer vessel wall is limited in parts due to the stiffness of the device.

The potential role of the endothelium in vascular remodelling after balloon injury has been discussed.^38^ Langille and O′Donnel demonstrated that a structural reduction in vessel size induced by a long‐term decrease in blood flow is dependent on an intact endothelium.^39^ On the other hand endothelium‐derived relaxing factor NO (EDRF‐NO) is involved in the adaptive enlargement of the vessel in response to increased blood flow.^40^ Measurements of EDRF‐NO levels following balloon injury in porcine coronary arteries demonstrated a decreased production of EDRF‐NO.^41^ As EDRF‐NO is a potent inhibitor of VSMC growth, the PCI‐induced damage of the endothelium is suggested to influence neointimal hyperplasia as well as the development of restenosis.

## 2 THE PROCESS OF RE‐ENDOTHELIALIZATION AFTER PTCA/STENT DEPLOYMENT

Arterial healing after denudation involves regrowth of the endothelium from remaining endothelial cells within the treated segment, from proximal and distal to the lesion as well as from side‐branch ostia.^42^ Circulating endothelial progenitor cells (EPCs) might also contribute to re‐endothelialization.^43^ The process begins within the first 24 hours after arterial denudation.^44^ A breakpoint of re‐endothelialization was observed at 6‐10 weeks in several animal models.^44^ In humans however there is limited information on the time‐course of re‐endothelialization following PCI.^23^

Delayed endothelial recovery has been identified as one of the major contributing factors of late stent thrombosis at autopsy.^45^, ^46^ The risk of thrombosis is substantially increased in stents with >30% uncovered struts compared to stents with complete coverage.^46^ Even beyond 1 year after implantation uncovered stent struts were identified in first‐generation sirolimus‐ and paclitaxel‐eluting stents, especially under high‐risk implantation conditions like acute myocardial infarction, bifurcation and ostial lesions, lesions in bypass grafts, lesions of the left main artery, chronic total occlusions (CTO), long lesions (>30 mm), and in‐stent restenosis.^47^, ^48^ Delayed arterial healing has also been observed in stents penetrating into the necrotic cores of atherosclerotic plaques and overlapping stents.^49^, ^50^

The biological factors controlling the re‐endothelialization process have not been completely elucidated. Both vascular endothelial growth factor (VEGF) and fibroblast growth factor (FGF) represent growth factors for endothelial cells whereas FGF also has trophic effects on VSMCs. Balloon injury induces a release of FGF and an increased expression of FGF mRNA in endothelial cells and VSMCs.^51^ Similarly, an increased expression of VEGF mRNA in rats could be observed.^52^ Studies performed by Lam et al in humans undergoing PTCA showed increased levels of circulating FGF, VEGF, and tumor growth factor b1 (TGF‐b1), suggesting an operative role of these factors in re‐endothelialization in humans.^53^

## 3 THE IMPACT OF STENT DESIGN ON ENDOTHELIAL REGROWTH

Today, a broad variety of stents is available. There have been significant developments concerning the design of stent platforms as well as the stent coatings including novel polymers, polymer‐free stents and bioresorbable stents. The endothelial recovery after stent implantation is influenced significantly by the stent design. The protrusion of stent struts leads to perturbations in the local flow patterns notably to the development of small regions with disturbed shear stress between the stent struts.^54^ Alterations in shear stress and blood flow dynamics are known to impact endothelial growth.^55^ In an experimental setting with flow and shear conditions similar to human arteries, the endothelial cell coverage area and migration was found to depend on object thickness and significantly decreased in objects with 75 μm thickness or greater.^56^ Relating to coronary stents, improved re‐endothelialization was demonstrated in newer generation stents with lower strut thicknesses.^57^ In line with that, re‐endothelialization was delayed in novel but comparably thick‐strut bioabsorbable stents as compared with thin‐strut everolimus‐eluting stents in a study of Koppara et al who performed stent implantation into iliofemoral arteries in a healthy rabbit model with contemporary stents used in clinical practice.^58^ Clinical significance was proven in the randomized multicentre Strut Thickness Effect on Restenosis Outcome” (ISAR‐STEREO)‐Trial demonstrating a significant reduction of angiographic and clinical restenosis after coronary artery stenting with thinner‐strut devices.^59^, ^60^

In addition to the strut thickness also the shape of the struts influences re‐endothelialization process by changes in local vascular flow conditions. Non‐streamlined stent struts promote flow separation in the regions proximal and distal to the stent strut. High shear at the edges of the struts can activate platelets through the release of thromboxane A_2_, whereas areas of low shear rates adjacent to non‐streamlined struts are associated with inhibition of re‐endothelialization, potentially enabling procoagulant and pro‐inflammatory elements to accumulate which contribute to thrombus formation.^54^ In contrast, streamlined strut geometry reduces flow separation and high shear peak, resulting in rapid re‐endothelialization and inhibition of platelet activation. Furthermore the areas affected by low shear stress are smaller adjacent to streamlined struts, contributing to a rapid re‐endothelialization.^54^

## 4 THE IMPACT OF STENTS ON ENDOTHELIAL FUNCTION

Angioplasty and stent implantation cause denudation of the endothelium. The regeneration and regrowth of the denuded endothelium originates from the remaining endothelial cells and uninjured segments at the stent edges and from side‐branch ostia. However, vessels that need to undergo an interventional revascularization process usually are affected by atherosclerosis. The endothelium of atherosclerotic vessels is dysfunctional a priori, characterized by the impairment of vasomotion, disruption of the hemostatic balance, and a pro‐inflammatory milieu.^20^ Thus, the regenerated endothelium after stent placement is found to be inadequate in terms of both barrier integrity and functionality with impaired endothelium‐dependent vasodilation and increased permeability.^61^ Concerning the vasomotor functions the rigid stent prevents relaxation and constriction of the artery. The production and release of bioactive substances in stented vessel segments are affected, resulting in limited ability to respond sufficiently to changes in the physical, chemical, and humoral environment.

Notably both the barrier integrity and the extent of dysfunctionality depend on the presence or absence of antiproliferative stent coverage. The majority of the currently available drug‐eluting stents release the mammalian target of rapamycin (mTOR) inhibitor sirolimus or one of its analogues (everolimus, zotarolimus, or biolimus). The mTOR pathway has an important role in modulating cell division in response to mitogenic stimuli by the control of protein synthesis mediated through formation of the TORC 1 and 2 complexes. Therapeutically the inhibition of mTOR and its target TORC inhibits the proliferation of smooth muscle cells (SMCs) in order to prevent restenosis. However, particularly in endothelial cells, TORC 1 and 2 components contribute to processes required for endothelial regrowth, function, and survival. TORC 1 influences the translation and activity of hypoxia‐inducible factor α, the most potent upregulator of VEGF, which is closely linked to the vascular healing process.^52^ TORC 2 regulates endothelial proliferation, migration and survival, and activation of eNOS, resulting in increased NO‐levels, so the simultaneous inhibition of TORC 1 and 2 is likely to have consequences to the regeneration process of the endothelium.^62^, ^63^ On this note, preclinical studies in rabbit models of atherosclerosis suggested that not only endothelial regrowth but also endothelial competence is more impaired within drug‐eluting stents than within bare‐metal stents.^45^ With regard to the synthetic functions of endothelial cells, the expression of the antithrombotic cofactor thrombomodulin was absent, or reduced in endothelia after implantation of DES compared to BMS.^58^ Thrombomodulin levels generally were lower in stented regions when compared to unstented regions.

The competence of the regenerated endothelium however seems to be better with newer‐generation than with first‐generation drug‐eluting stents. A comparison of the expression of eNOS following implantation of sirolimus‐, everolimus‐ and zotarolimus‐eluting stents in a rabbit model of atherosclerosis demonstrated a less‐delayed healing and greater expression of eNOS after deposition of a zotarolimus‐eluting stent.^64^

Intercellular junctions maintain the barrier integrity. Transmission electron microscopy of rabbit iliac arteries after implantation of DES demonstrated poor cell‐to‐cell contact and monocyte attachment.^65^ Koppara et al performed the implantation of comparator polymer‐based drug‐eluting stents in rabbit iliac arteries.^58^ Despite apparent endothelial recovery in scanning electron micrographs 28 days after placement of first‐generation sirolimus and paclitaxel‐eluting stents a reduction of the transmembrane protein platelet/endothelial cell adhesion molecule 1 (PECAM‐1, also known as CD 31) at endothelial cell borders could be observed. This observation indicates a delay in maturation or increased turnover of endothelial cells.

## 5 STRATEGIES TO PROMOTE RE‐ENDOTHELIALIZATION AFTER STENT IMPLANTATION

Incomplete re‐endothelialization has been demonstrated particularly after the implantation of drug‐eluting stents due to the unselective antiproliferative effect of the drugs on both VSMCs and endothelial cells. It has been suggested that DES should ideally have a selective antiproliferative effect on VSMCs but be inert towards EC or better still promote their proliferation. The latter might be achieved by targeting factors influencing VSMC and EC growth. In this regard the impact of the administration of growth factors as well as the capture of EPCs have been investigated. In an effort to promote and accelerate the process of re‐endothelialization after stent implantation, novel stent designs have been developed, summarized as pro‐healing stents.

Van Belle et al demonstrated a promotion of re‐endothelialization after stent implantation by local administration of VEGF in a rabbit iliac model.^66^ Seven days after stent implantation, a near complete re‐endothelialization of the stented vessel was detected whereas the endothelial coverage of the stented arterial segment without VEGF was only 30%.^67^ Based on these findings, growth‐factor delivering stents have been developed, however they failed to promote re‐endothelialization in vivo and increased neointimal proliferation instead.^68^

Another strategy to improve re‐endothelialization is followed by capturing EPCs from the blood flow. EPC capture has been attempted by coating stents with antibodies that target EPC markers such as anti‐CD133, anti‐VE‐cadherin (anti‐CD 144), and anti‐CD34.^69^ Coating of stents with anti‐CD133 antibodies did not influence re‐endothelialization or neointimal thickening in a porcine model.^70^ VE‐cadherin is an adhesion molecule participating in the formation and maintenance of endothelial cell‐cell communication. It is specifically expressed in vascular endothelial cells. Coating of stents with anti‐VE‐cadherin antibodies could accelerate re‐endothelialization and reduce neointimal formation in a rabbit model.^71^ Among EPC subsets, outgrowth endothelial cells (OECs) preferentially express VE‐cadherin, and exhibit greater vasculogenic activity with more rapid proliferation and more active migration.^72^ Therefore capturing OECs more selectively with anti‐VE‐cadherin antibody could be responsible for a more efficient re‐endothelialization and anti‐thrombogenicity of anti‐VE‐cadherin antibody‐coated stents in animal models.^73^

The Genous^®^ (OrbusNeich) stent, coated with anti‐CD34 antibodies to capture EPCs, was the first device of its kind to be evaluated in humans. In preclinical porcine studies the early re‐endothelialization process was enhanced without affecting neointimal proliferation.^69^ Likewise, in humans neointimal thickness was not reduced as confirmed by angiographic and intravascular ultrasound follow‐up.^74^ Compared to first‐generation paclitaxel‐eluting stents, the TRIAS trial showed no significant difference in mortality, myocardial infarction, and target vessel revascularization at 2 years.^75^ As CD34 antibodies are not specific to EPCs, an attraction of other hematopoetic stem cells such as smooth muscle progenitor cells as well as inflammatory cells might cause an increase in neointimal proliferation. To overcome this issue, the Combo^®^ (OrbusNeich) stent combines CD34 antibody‐mediated EPC capturing on the luminal surface and a sirolimus‐eluting biodegradable polymer on abluminal surface. Reduced neointimal proliferation and accelerated re‐endothelialization were demonstrated in a porcine model.^76^ The first‐in‐men REMEDEE (Randomized Evaluation of an Abluminal sirolimus coated Bio‐Engineered Stent) study revealed safety and non‐inferiority to everolimus‐eluting Xience V stent.^77^ However, in the prevention of in‐stent restenosis in complex lesions the dual endothelial capturing stent technology is less effective compared with drug‐eluting stents^78^; further data is to be expected.

A combination of the administration of growth factors and the concept of EPC capture is followed by Song et al who performed in vitro studies with stainless metallic steel coated with VEGF and anti‐CD34 antibody.^79^ With co‐coating of VEGF and anti‐CD34 the differentiation of EPCs in vitro was enhanced compared to single VEGF coating and bare metal, so simultaneously coating stents with VEGF and anti‐CD34 antibody might be a novel research direction for facilitating re‐endothelialization in order to reduce restenosis after stent implantation.

Cyclic‐RGD (Arg‐Gly‐Asp)‐peptide has been identified as a recognition sequence for integrins and thus represents a binding motif specifically attracting endothelial progenitor cells.^80^ The use of RGD‐containing peptides on surfaces is known to enhance the adhesion, growth, and spreading of endothelial cells.^81^ A novel stent coated with cRGD was investigated earlier by our group.^82^ The cRGD coating clearly supported the outgrowth, recruitment, and migration of EPCs in vitro. Scanning electron microscopy indicated enhanced endothelial coverage on cRGD‐loaded stents at 4 weeks in a porcine model that might contribute to a reduction of restenosis seen at 12 weeks. To achieve a specific attraction of OECs, a combinational stent coating with certain signalling molecules has been investigated.^83^ Bio‐functionalization with RGD/CXCL1 effectively supported the adhesion and proliferation of OECs in vitro. After implantation of RGD/CXCL1‐coated stents in carotid arteries of apoliproprotein E^−/−^ mice an accelerated re‐endothelialization by supporting the adhesion and proliferation of EPCs, especially OECs, was detected, so RGD/CXCL1 coating of stents may help to diminish the risks of stent thrombosis and restenosis.

However, patients with cardiovascular diseases often have poor or dysfunctional EPCs. The treatment with statins has been associated with an increased number and survival of EPCs in patients with cardiovascular diseases.^74^

## 6 STRATEGIES TO IMPROVE ENDOTHELIAL FUNCTION IN STENTED CORONARY ARTERY SEGMENTS

Strategies enhancing the process of re‐endothelialization in stented coronary arteries do not necessarily result in reconstitution of all endothelial functions. To overcome the issue of incomplete and dysfunctional re‐endothelialization even immediately after denudation, Andukuri et al developed a bioinspired multifunctional nanomatrix mimicking the endothelial surface characteristics by containing cell‐adhesion ligands and NO‐donors.^84^ The recruitment and differentiation of EPCs towards an endothelial lineage was improved, however the device has not been tested in preclinical models.

ACE inhibitors have been described as beneficial on intimal hyperplasia following balloon angioplasty.^85^ Besides the inhibition of conversion of angiotensin I to the vasoconstrictive agent angiotensin II, ACE inhibitors are known to inhibit kinin hydrolysis with stimulation of NO release.^86^ The administration of ACE inhibitors has demonstrated an inhibition of neointimal hyperplasia in different animal models of balloon denudation.^85^ Simultaneously, van Belle et al have demonstrated an enhancement of endothelial regrowth as well as an improved endothelial function in a rabbit model of balloon injury.^87^ Interestingly, scanning electron microscopy analysis showed a morphological change towards recovery of a spindle shape of the endothelial cells, which has been associated with functional recovery of endothelial cells. However with regard to the progression of restenosis after stent implantation in humans, the administration of ACE inhibitors neither improved the ischemic threshold nor reduced the need for new revascularization procedures.^88^ Studies that evaluated a possible anti‐atherosclerotic effect of ACE inhibitors (including large randomized trials) have generally been negative.^88^

Exercise training is one of the key factors in primary and secondary prevention of cardiovascular diseases. Indolfi et al demonstrated that exercise training increases eNOS expression and activity resulting in an increased bioavailability of nitric oxide in the vascular wall of rats.^89^ Histological assessment after balloon angioplasty and stent implantation in rats undergoing a daily training program demonstrated a complete re‐endothelialization of injured vessels with increased eNOS activity, and reduced platelet aggregation.^89^ However, the implantation of conventional metallic stents is associated with an impairment of vasomotion that in parts results from the caging foreign material, thus the vasodilative effect of NO in stented artery segments is limited.

A promising strategy towards restoration of a normal vessel function is the development of fully bioresorbable scaffolds providing mechanical stability for a finite period after PCI and then being gradually resorbed leaving the vessel free of any foreign material. Today, there are several bioresorbable scaffolds at various stages of development, based on either metallic alloys or polymers. However, all bioresorbable scaffolds are characterized by thick stent struts when compared to second generation DES.^28^ Considering the negative impact of strut thickness to re‐endothelialization and thrombogenicity, Koppara et al demonstrated a delayed re‐endothelialization and an increased acute thrombogenicity after implantation of fully bioabsorbable stents compared to thin strut second generation DES and BMS in the rabbit iliofemoral artery model.^58^ Absorb^®^ bioresorbable vascular scaffold (BVS) achieved a CE mark in 2011 as the first drug eluting fully bioresorbable scaffold. Imaging studies in porcine coronary arteries supported a restoration of cyclic pulsatility at the device site at 6 months after implantation.^90^ Fully restored vasomotion was observed at 12 months, and progressive lumen gain with plaque regression was documented between 2 and 5 years both in porcine coronary arteries and in humans.^91^, ^92^ The pathophysiology underlying the observed lumen gain and plaque regression is as yet unknown and remains an area of research interest. Remarkably, subsequent to the loss of scaffolding physiological fluid dynamics could be restored, including a return of a more normalized arterial flow, shear stress, and cyclic forces. As these forces influence the anatomy and function of endothelial and smooth muscle cells and regulate vascular remodelling, the effects of lumen gain and restoration of pulsatility following BVS‐Implantation might be based on more physiological flow conditions.^26^ Since its commercial launch the BVS has been studied in registries^93^, ^94^ and randomized trials, but long‐term follow‐up is required.^95^ Within 1 year there was no significant difference between the BVS and a thin strut second generation DES (Xience) in rates of cardiac death, target‐vessel revascularization or target‐vessel myocardial infarction.^95^ However, stent thrombosis occurred more frequently following implantation of BVS within 1 year, but it has to be pointed out that the studies were underpowered to this event. Nevertheless the finding of an increased rate of stent thrombosis might be related to an incomplete re‐endothelialization process, as previous animal studies suggested.^58^, ^95^ Improvements in stent design notably in terms of strut thickness might be an area for future research.

## 7 CONCLUSION AND FUTURE PERSPECTIVES

Catheter‐based interventional coronary revascularization procedures cause vascular injury including endothelial denudation. Compared to bare metal stents, drug‐eluting stents have achieved dramatically reduced restenosis rates. However, the antiproliferative drugs lack selectivity with respect to the targeted cell types. So not only the proliferation of VSMCs, underlying neointimal formation, is inhibited, but also the endothelial repair is compromised. Incomplete re‐endothelialization after implantation of stents has been identified as the primary cause of an increased risk of late and very late stent thrombosis. The regenerated endothelium in stented regions however is inadequate with regard to barrier, antithrombotic, and vasodilative functions, represented by poorly formed cell‐to‐cell junctions and reduced expression of antithrombotic molecules and eNOS. Additionally, stent‐induced disturbances of blood flow contribute to complex alterations in shear stress, resulting in increased thrombogenicity around the stent struts and impairment of re‐endothelialization. A complete regeneration of vascular endothelium prohibits neointimal thickening and hyperplasia and finally prevents restenosis. The long‐term health of the vessel wall depends on a successful restoration of a competent endothelium. Efforts have been made to promote re‐endothelialization and to improve endothelial functions after stent implantation by changes in stent design and coating. Future studies must address the long‐term safety of both new generations of DES and fully bioresorbable scaffolds aiming at the maintenance of a competent and functional endothelium in order to reduce stent thrombosis and restenosis rates.

## CONFLICT OF INTEREST

The authors confirm that there are no conflicts of interest.
